# The Effect of the Antidepressant Citalopram on the Bioconcentration and Biomarker Response of *Daphnia magna* at Environmentally Relevant Concentrations

**DOI:** 10.3390/toxics13070532

**Published:** 2025-06-25

**Authors:** Haohan Yang, Jiacheng Tan, Hanyu Jiang, Hao Xing, Jingnan Zhang, Dexin Kong, Zhuoyu Chen, Linghui Kong

**Affiliations:** College of Environmental Science and Engineering, Yangzhou University, Yangzhou 225127, China; 231601213@stu.yzu.edu.cn (J.T.); 231602108@stu.yzu.edu.cn (H.J.); mz120241359@stu.yzu.edu.cn (H.X.); 231601220@stu.yzu.edu.cn (J.Z.); 231601208@stu.yzu.edu.cn (D.K.); mz120231301@stu.yzu.edu.cn (Z.C.); mz120231312@stu.yzu.edu.cn (L.K.)

**Keywords:** bioconcentration, phenotypic effects, biomarker responses, citalopram, *Daphnia magna*

## Abstract

The widespread use and pseudo-persistent occurrence of the antidepressant citalopram (CIT) could pose a potential ecological risk in the aquatic environment. The message about the bioconcentration and sensitive biomarker identification of CIT at the environmentally relevant concentrations is limited. In this study, an integral evaluation of the phenotypic and biochemical effects of CIT on *Daphnia magna* (*D. magna*) was conducted at 0.5 and 10 µg/L. The biomarker screening includes energy metabolism, phototactic behavior, feeding dysfunction, and antioxidant stress responses. The carbohydrate, lipid, and protein content was determined using the assay of anthrone with glucose as standard, thiophosphorate-Vaniline with cholesterol as standard, and Coomassie brilliant blue with serum albumin as standard, respectively. The results showed the bioconcentration equilibrium of CIT reached at the exposure duration of 48 h during the uptake process. At the exposure concentrations of 0.5 and 10 µg/L, the bioconcentration factor of CIT was 571.2 and 67.4 L/kg, respectively. Both protein and lipid content significantly increased at 0.5 µg/L with a 1.78-fold elevation in total energy. Comparatively, the lipid content showed a significant increase at 10 µg/L, while the available total energy rose by 1.25-fold relative to the control group. The phototactic behavior of *D. magna* exposed to 0.5 µg/L CIT was markedly reduced at 48 h relative to control. In contrast, a significant decrease in phototaxis was observed after 6 h and then a significant increase at 12 h with a continuously obvious decline at 10 µg/L. The filtration rates were increased by 32% compared to controls at 0.5 µg/L, while the stimulatory effects disappeared at 10 µg/L. With regarding to the antioxidant enzyme activities, CIT exposure significantly inhibited the catalase activity both at 0.5 and 10 µg/L, while the glutathione S-transferase activity was obviously induced at 0.5 µg/L and inhibited at 10 µg/L. The expression level of *18s* gene was significantly decreased at 10 µg/L. Only the *gst* gene expression level was significantly increased at 0.5 µg/L, while the *18s* and *cat* gene expression level was obviously inhibited and induced at 10 µg/L. Comprehensively, the responses of the phenotypic traits and energy metabolism of *D. magna* at various environmental concentrations were sensitive for CIT. This study provided basic data for the risk estimation of CIT in the real freshwater environment.

## 1. Introduction

Selective serotonin reuptake inhibitors (SSRIs), an antidepressant prescribed for psychiatric disorders, involve the primarily selective inhibition of serotonin (5-HT) reuptake at presynaptic neuronal membranes, thereby elevating extracellular serotonin concentrations within the synaptic cleft and potentiating serotonergic neurotransmission [1]. Recently, SSRIs have been considered to be pseudo-persistent and omnipresent in aquatic environments due to their widespread application and incomplete removal by wastewater treatment plants. The ubiquity of SSRIs in receiving waters is found with a detected concentration of even up to µg/L levels [2]. As a result, the SSRI residues are detected in non-target aquatic wildlife at various trophic levels [3]. A series of studies have also discovered the biological effects of SSRIs in laboratory investigation, such as behavior alteration, the disorder of energy metabolism, and oxidative stress [4,5]. It is not surprising that more information about the potential environmental risk of the neuroactive compounds should be discovered.

**Figure 1:**
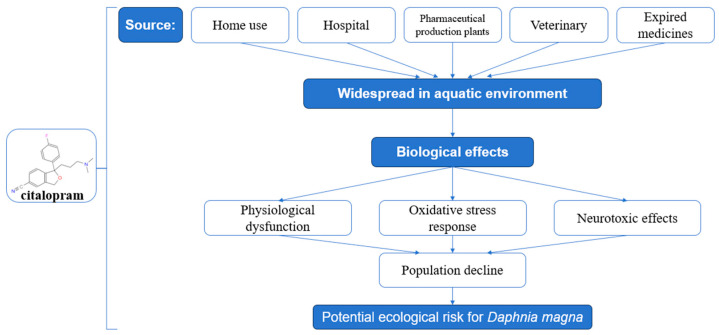
Harmful processes of citalopram (CIT) to *Daphnia magna* in water bodies.

Among SSRIs, citalopram (CIT) has raised significant environmental concerns due to its high persistence and bioaccumulation potential. As shown in Figure 1, the process of harmful effects exerted by aqueous citalopram (CIT) on Daphnia magna can be clearly observed. For instance, CIT has been detected at 76 µg/L in surface water and 1.4 µg/L in well water in India [6], while high detection rates (>80%) were observed in municipal wastewater treatment plants in the United States [7]. In Canadian and Norwegian urban wastewater, CIT concentrations have reached 3.4–223 µg/L and 9.2–612 µg/L, respectively [8]. Although fluoxetine and sertraline exhibit relatively high bioaccumulation potential in invertebrates with bioconcentration factors (BCF) of 185,900 and 1173 L/kg [9,10], the uptake and elimination processes with CIT remain poorly quantified. Furthermore, the feeding behavior of organisms exerts significant influences on their intrinsic energy metabolism, while potentially modulating the vital activities of adjacent populations [11]. Notably, exposure to psychiatric drugs such as carbamazepine, diazepam, and propranolol at environmentally relevant low concentrations significantly enhanced negative phototaxis in *Daphnia magna* (*D. magna*), whereas higher concentrations paradoxically attenuated this behavioral response [12].

However, the sensitive biological indicators of CIT have not yet been explored at the environmentally relevant concentration. For example, the behavioral endpoints such as phototaxis inhibition and feeding suppression have been characterized as biomarkers for ecotoxicology [13]; the mechanistic links between CIT-induced neurotoxicity, energy homeostasis, and behavioral alterations have not yet been elucidated [14]. Bioaccumulation is the ability to quantify the environmental persistence and trophic transfer potential of CIT, while available energy patterns could reveal its metabolic adaptations (stress protein synthesis and lipid catabolism). Concurrently, phototactic and feeding behaviors have been assessed to link neurotoxic effects with energy metabolic shifts, and the antioxidant profiling would map the oxidative stress and compensatory antioxidant interactions. The integration of novel biomarkers could systematically advance the ecotoxicological risk assessment of CIT.

In fact, a series of the biological effects of CIT have been found in aquatic organisms, such as neurotoxicity, reproductive and developmental toxicity, and immune suppression. For example, the significant inhibition of acetylcholinesterase (AChE) activity accompanied by locomotor coordination deficits and circadian rhythm disruption were observed in *Danio rerio* at CIT exposure concentrations of 10–100 μg/L [15,16]. Markers of oxidative stress such as reactive oxygen species (ROS) and malondialdehyde (MDA) levels may increase, while the activity of antioxidant enzymes such as superoxide dismutase (SOD) may decrease. The chronic exposure experiments (150 days) of *Danio rerio* revealed that CIT resulted in locomotor dysfunction and memory impairment in F1 offspring, concomitant with the dysregulated expression of neurodevelopmental genes [17]. Similarly, the obvious reduction in swimming activity frequency and a 24 h delay in ovisac development were found at a CIT concentration of 10 μg/L in *Daphnia magna* [18]. Thus, it is necessary to explore the ecological toxicological effects of CIT at environmental-related concentrations.

As an indispensable part of the food web in the aquatic ecosystem, *Daphnia magna* (*D. magna*) is a premier model organism in ecotoxicology due to its high sensitivity to contaminants. Collectively, its short life cycle and parthenogenetic reproduction facilitate rapid, cost-effective, multigenerational, and chronic toxicity assays under standardized laboratory conditions. The present study’s objective is to investigate the biological influence of CIT at environment-related concentrations using *D. magna* as the test animal. The bioconcentration potential, available energy, feeding and phototactic behaviors, antioxidant enzyme activities, and related gene expression were selected as the biomarkers in *D. magna*. Based on the dynamic equilibrium of CIT during the uptake process, the metabolic profiles (proteins, lipids, carbohydrates) and antioxidant enzyme activities (SOD, CAT, GST) were quantified to elucidate stress adaptation mechanisms. Concurrently, the phototactic and feeding behaviors are assessed to link neurotoxic effects with energy metabolic shifts. This study establishes a predictive framework for ecological risks associated with SSRIs contamination, underscoring the utility of *D. magna* as a biosensor for neurotoxic effects.

## 2. Materials and Methods

### 2.1. Chemicals and Reagents

Citalopram hydrobromide (CAS 59729-32-7, analytical standard grade, ≥98% purity) was acquired from J&K Scientific Co., Ltd. (Shanghai, China). Enzyme activity assay kits for superoxide dismutase (SOD), catalase (CAT), glutathione S-transferases (GST), and protein quantification were procured from Nanjing Jiancheng Bioengineering Institute (Nanjing, China). Chromatographic-grade solvents (acetonitrile, methanol) were sourced from Merck Serono Co., Ltd. (Darmstadt, Germany), with chemical stocks prepared in anhydrous acetonitrile (HPLC ≥ 99.9%) and maintained at −20 °C in amber vials.

### 2.2. Experimental Setup

The exposure concentrations of two treatments (0.5 and 10 µg/L) were selected according to the pollution levels of CIT in the actual water body. It has been found that the CIT concentration in surface water and in urban receiving water was in the range of several hundred ng/L [19] and several dozen µg/L [6], respectively. Therefore, these two concentrations were selected as the exposure concentrations as typical examples. At the start of the exposure, 21-day daphnids were randomly selected and transferred into beakers containing 1000 mL of exposure medium and 240 individuals [20]. Then, sampling was performed at 0, 3, 6, 12, 24, 36, and 48 h post exposure. The exposure duration selected was based on our pre-experiment results [21] and previous studies of Ding et al. [22], which state that 48 h is long enough to ensure a steady state of CIT accumulation in *D. magna*. Approximately ten organisms were collected at each time point using a glass pipette and rinsed with fresh culture medium in a Petri dish. Following filter paper desiccation, samples were aliquoted into 2 mL microcentrifuge tubes. The pooled wet weight was weighed using a microbalance (accuracy: ±0.01 mg), and samples were stored at −80 °C. The exposure solution was changed every 24 h. Three independent replicates were implemented per exposure level. Methanol concentration was maintained below 0.1 mL/L in the test medium. At the end of exposure, the remaining animals were taken out for the determination of behavioral responses (20 individuals), available energy (50 individuals), antioxidant enzyme activities (50 individuals), and gene expression (50 individuals).

### 2.3. Chemical and Biochemical Analysis

#### 2.3.1. Extraction and Analysis of CIT

The extraction and quality control of CIT determination in *D. magna* are mainly based on our previous studies [21,23], in which the recovery rate of the method was in the range of 72.3–81.2%. The biota samples were homogenized three times with 5 mL of acetonitrile. Then, the homogenate was transferred into 10 mL centrifuge tubes. Subsequent processing included 30 min ultrasonication followed by centrifugation at 4000× *g* for 5 min to achieve phase separation. The supernatant was collected into a clean 20 mL centrifuge tube, and two replicates of the extraction workflow were performed. The combined extracts were evaporated to dryness under a nitrogen flow and reconstituted in 1 mL of acetonitrile. The quantification of CIT was performed using a Waters Acquity^TM^ UPLC-MS/MS System (Waters, Milford, MA, USA) with a Xevo^TM^ Triple Quadrupole MS (Waters Corp., Milford, MA, USA) mass spectrometer with an electrometer ionization (ESI) source. Detailed information on the sample analysis can be found in Supplementary Information Text S1, Tables S1 and S2.

#### 2.3.2. Available Energy Analysis

Pre-frozen daphnid samples were homogenized in 0.1 M potassium dihydrogen phosphate buffer (pH 7.4) and centrifuged at 4 °C for 15 min (15,000× *g*). Using chloroform, we extracted total lipids and carbohydrates from tissue in the homogenates:methanol ratio of 2:1 (*v*/*v*). The carbohydrate was measured using an anthrone assay based on glucose [24]. The bottom phase was used to determine the total lipid content using the cholesterol-standard thiophosphorate-Vaniline test [25]. Proteins were quantified using the approach of Gómez-Canela C with serum albumin as the standard [26]. The total energy (Ea) for growth and reproduction is calculated by adding the total content of proteins, lipids, and carbohydrates, and then converting each energy component into an energy equivalent using the enthalpy of combustion (24 kJ/g protein, 39.5 kJ/g lipids, and 17.5 kJ/g carbohydrates) [27].

#### 2.3.3. Antioxidant Enzyme Activity Analysis

Under ice-cold conditions, pre-frozen Daphnid samples were homogenized in 0.1 M potassium dihydrogen phosphate buffer (pH 7.4), and then the samples were centrifuged at 4 °C for 15 min at 15,000× *g*. The resulting supernatant was marked for biochemical assays, with the immediate determination of the enzyme activities of SOD, CAT, and GST making accompanying kits. All biospecimen pools underwent triplicate catalytic activity determinations via standardized methods and enzyme protocols. The enzyme activities in all the samples were analyzed in triplicate for each pool using the microplate reader Multiskan FC 100 (ThermoFisher Scientific, Waltham, MA, USA). SOD activity was determined by its inhibitory capacity on the reduction in cytochrome c in the presence of a hypoxanthine/xanthine oxidase O^2−^-generating system. The method was employed to assay CAT activity in the previous study, which measures the consumption of substrate (H_2_O_2_) through a decrease in absorbance at 240 nm [21]. GST activity was assayed using the method by Sharma [28], which measures the conjugation of glutathione (GSH) with 1-chloro-2,4-dinitrobenzene (CDNB) at 340 nm. Protein content was measured using the method in the previous study, with serum albumin as the standard [26]. The activity units of these three enzymes are expressed as nmol/mg total protein/min.

### 2.4. Behavioral Analysis

#### 2.4.1. Phototactic Behavior

After exposure to specific concentrations of CIT under the required conditions, pre-acclimated daphnids (5 individuals per replicate) were transferred to a small glass column (25 cm height, 5 cm internal cross-section) filled with ASTM hard water alone or with ASTM hard water spiked with the desire test concentration, with a 50 W compact fluorescent light source positioned at the top. After a 10 min adaptation period (five minutes each during the water column and the darkness), the light source was lit, and then the positions were recorded. The phototactic index (I) was counted as (U − L)/(U + M + L), where U, M, and L represent the numbers of animal observations in the upper, middle, and lower compartments, respectively [12].

#### 2.4.2. Feeding Behavior

After 48 h of exposure duration, 20 individuals for each group were obtained for the feeding behavior test. Each treatment contained triplicate beakers with *D. magna* in 100 mL exposure medium with *Scenedesmus obliquus* suspension at 1 × 10^6^ cells/mL. The control group contained only an algal solution to account for background algal growth. All procedures were performed in darkness at 20 ± 1 °C with gentle shaking every 30 min to maintain algal suspension. Algal concentrations were quantified initially (*C*_0_) and after 5 h (*C_t_*) using a Neubauer hemocytometer (0.1 mm depth) at 400× magnification. Filtration (*F*) and ingestion (*I*) rates were determined following established protocols with modifications [29]. The equations below account for algal growth in controls:

(1) $$F=\frac{V}{n}\times \frac{\ln C_{0}-\ln C_{t}}{t}-A$$

(2) $$A=\frac{\ln C_{0}-\ln C_{t}^{\prime }}{t}$$

(3) $$I=F\times \sqrt{C_{0}\cdot C_{t}}$$

where *V* represents volume (µL), *n* is the organism count, *t* is exposure duration (h), and *A* is the algal growth correction factor from controls.

### 2.5. Gene Transcription Analysis

The total RNA in *D. magna* specimens was achieved through guanidinium thiocyanate-phenol-chloroform extraction using Trizol reagent (Invitrogen, Carlsbad, CA, USA) following the descriptions. The total RNA was then reversely transcribed to complementary DNA (cDNA) using M-MuLV reverse transcriptase (Promega, Madison, WI, USA) and oligo (dT) 15 primer. Quantitative real-time PCR reactions were conducted using a fluorescent quantitative detection system (FQD-48A) (BIOER, Hangzhou, China), and the conditions were as follows: 95 °C for 3 min, 40 cycles at 95 °C for 10 s, and 60 °C for 60 s followed by melting curve analysis to confirm single PCR products. Threshold cycle (Ct) values were used to express the qRT-PCR data of sod, cat, and glutathione S-transferase (*gst*). The mRNA expression levels were analyzed using the 2^−△△Ct^ method. Gene-specific primers were designed for all genes according to NCBI (http://www.ncbi.nlm.nih.gov/), and the β-actin gene served as a control. Further specifics on the process mentioned earlier and the primer message can be found in Table 1. The *18s* gene was marked to use as an internal control, and the primers were the same as those described in a former study [30].

### 2.6. Integrated Biomarker Response Analysis

This study used phototaxis, carbohydrate, lipid, protein, ingestion rates, filtration rates, GST, SOD, CAT, *gst* gene, *cat* gene, and *18s* gene to count the biomarker reaction version 2 comprehensive index (IBR_v2_) results [31,32]. IBR_v2_ was counted following the ways described by Yang [31], with Sanchez amending it [32]. More information about IBR_v2_ can be located in the Support Information (Text S2).

### 2.7. Data Analysis

Error margins on CIT data points denote triplicate measurements’ standard deviation (SD), whereas biological indices are presented with standard error (SE) bars derived from triplicated experimental units. Inter-group variations were statistically evaluated through one-way analysis of variance (ANOVA) with Duncan’s multiple range test for post hoc comparisons at a *p* = 0.05 significance threshold. All computational procedures were executed using SPSS Statistics (Version 22.0; SPSS Inc., Chicago, IL, USA), with probability values below 0.05 constituting statistically significant differences.

## 3. Results and Discussion

### 3.1. Effect of CIT on the Bioconcentration of D. magna

**Figure 2:**
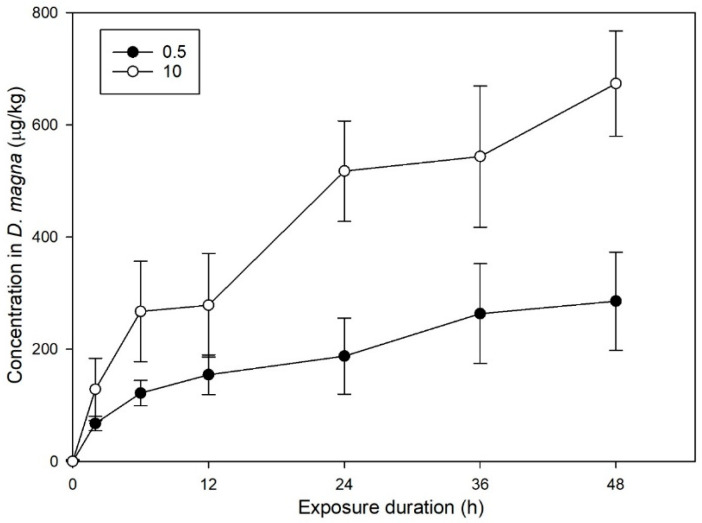
Concentration of CIT in *D. magna* exposed to nominal concentrations of 0.5 and 10 µg/L. Data are mean ± SD (n = 3).

As illustrated in Figure 2, CIT accumulation in *D. magna* exhibited time- and concentration-dependent trends, ultimately reaching a dynamic equilibrium. For the 0.5 µg/L group, accumulation accelerated during the initial 12 h and stabilized at 285.6 µg/kg by 48 h. Comparatively, rapid uptake occurred within the first 24 h at 10 µg/L, followed by a gradual plateau and yielding a steady-state concentration of 673.6 µg/kg after 48 h. Notably, CIT’s high bioaccumulation potential at environmentally relevant low concentrations aligns with its lipophilicity (log *K*_ow_ = 4.5) and lipid-binding properties in the cuticle of *D. magna* [33]. According to some studies, establishing a steady state during exposure may arise from growth dilution effects in the model organism and dynamic equilibrium between uptake and elimination processes. As evidenced by the absence of significant changes in the average body weight of *D. magna* throughout the experimental period, the observed steady state is predominantly attributed to the balance between uptake and elimination processes [34]. Once the aquatic environment is polluted by CIT, even at low concentrations, it can still lead to the accumulation of CIT in *D. magna*. Moreover, it was difficult for the residual CIT to be removed by *D. magna*, which affected their growth and reproduction [35]. This can also result in the accumulation of toxic substances during food chain transmission [36]. Such low-concentration/high-bioaccumulation characteristics may amplify ecological risks via trophic transfer (e.g., small fish to predatory fish), which is similar to findings of fluoxetine in daphnids [37].

Based on the experimental data, the bioconcentration factors (BCF) at 0.5 and 10 µg/L were calculated as 571.2 and 67.36 L/kg, respectively. The results suggest that CIT exhibits stronger bioaccumulation potential at environmentally relevant low concentrations. This may be attributed to metabolic regulatory mechanisms, as the active uptake of CIT might dominate at low concentrations, whereas detoxification processes (e.g., the suppression of GST activity) or enhanced elimination could reduce BCF at higher concentrations [9,34]. The high BCF (571.2 L/kg) at 0.5 µg/L implies that even trace levels of CIT (e.g., µg/L) may induce toxic effects on aquatic organisms like *D. magna* through bioaccumulation [35]. Furthermore, the persistence and biomagnification potential of CIT could amplify ecological risks via trophic transfer (e.g., to fish and predators) [34,36]. The bioaccumulation capacity of CIT decreases with increasing exposure concentration, and yet its high BCF at low levels poses potential risks to aquatic ecosystems. Future research should integrate field monitoring and food chain models to evaluate the long-term ecological impacts and synergistic effects of CIT with co-occurring contaminants.

### 3.2. Effect of CIT on the Energy Available to D. magna

**Figure 3:**
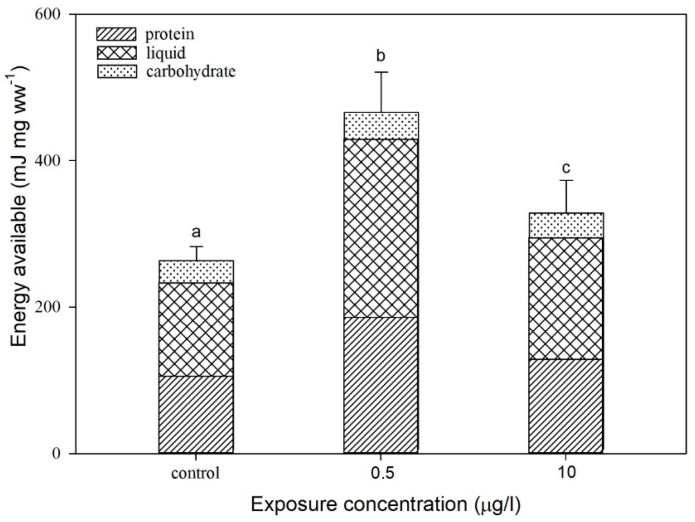
Energy available to *D. magna* at CIT exposure concentrations of 0.5 and 10 µg/L. The proportions are taken by total content of proteins (oblique), lipids (grids), and carbohydrates (dotted). Different letters indicate significant disparity from controls (*p* < 0.05). Data are mean ± SD (n = 3).

The protein and lipid contents under CIT exposure are presented in Figure 3. Both protein and lipid contents significantly increased at 0.5 µg/L with a 1.78-fold elevation in total energy. Comparatively, the lipid content showed a significant increase at 10 µg/L, while available total energy rose 1.25-fold relative to the control group. Likewise, analogous consequences were observed in former studies [21,27,38]. This phenomenon may be attributed to stress-induced physiological responses in *D. magna*, inducing the synthesis of stress proteins and thereby augmenting the protein content [30]. The dual roles of protein accumulation could indicate the immediate energy substrates via proteolytic pathways during acute stress phases, replenishing antioxidant enzyme pools to counteract CIT-induced oxidative damage [39,40]. Imbalanced lipid intake may disrupt growth and reproduction in *D. magna* [25]. To counteract energy deficits caused by CIT toxicity, *D. magna* likely maintains dynamic energy homeostasis through lipid metabolism [25,41]. Hence, a potential self-protective mechanism was activated at a low CIT concentration of 0.5 µg/L, characterized by a synthesis of stress-related proteins to repair and replenish energy reserves. In contrast, under a high CIT exposure of 10 µg/L, these adaptive mechanisms were suppressed, resulting in excessive energy expenditure and heightened lipid catabolism, thereby diminishing the total energy gain compared to low-concentration exposure [42]. Concentration-dependent energy modulation carries significant ecological implications. The enhanced energy reserves at 0.5 µg/L may temporarily increase stress tolerance but could impair population sustainability through resource diversion from reproduction [43]; organisms under chronic stress reallocate resources from reproduction to maintenance [44]. Conversely, the diminished energy gain at 10 µg/L indicates total metabolic exhaustion, and it may be attributed to ATP synthesis efficiency reduction [45]. These findings underscore the importance of considering non-monotonic dose responses in environmental risk assessments for neuroactive pharmaceuticals [46]. This mechanistic transition from adaptive hormesis to pathological disruption not only highlights the delicate balance between xenobiotic-induced stress responses and metabolic homeostasis in aquatic organisms, but also underscore the environmental risks of sublethal antidepressant exposure in aquatic ecosystems. In fact, 0.5 µg/L CIT significantly induced protein (+45%) and lipid (+32%) synthesis, with total energy increasing 1.78-fold, likely driven by heat shock protein (HSP) upregulation under stress [47]. HSPs not only facilitate protein repair, but also maintain energy homeostasis by regulating antioxidant enzyme activity. In contrast, the 10 µg/L group exhibited disrupted lipid metabolism; while total energy still increased 1.25-fold, lipid catabolism markers (e.g., acyl-CoA oxidase) rose 2.1-fold [48], suggesting that high-dose CIT activates peroxisome proliferator-activated receptor (PPAR) pathways to mobilize lipid reserves for energy crisis. This mirrors lipid metabolic disorders observed in zebrafish embryos exposed to SSRIs [49]. The stable carbohydrate content in both groups implies that *D. magna* prioritizes lipid over carbohydrate as an energy source during stress, likely due to the high energy demand of its planktonic lifestyle [50].

### 3.3. Effect of CIT on the Behavior Response of D. magna

#### 3.3.1. Phototactic Behavior

**Figure 4:**
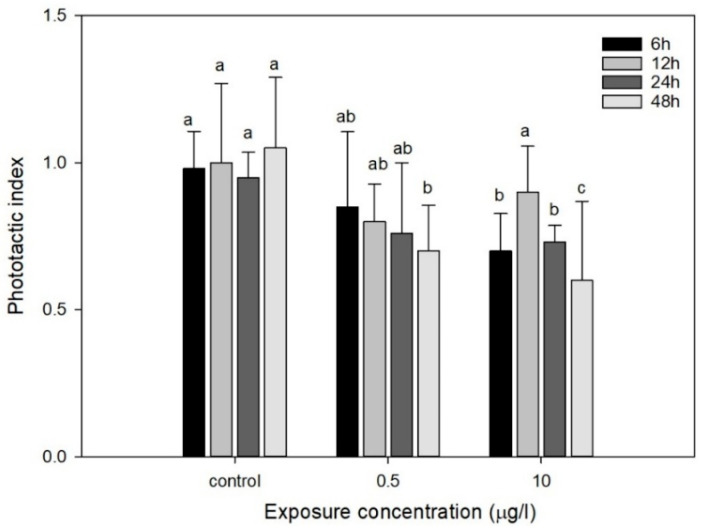
Effect of exposure time periods on the phototropism index of *D. magna* at concentrations of 0.5 and 10 µg/L. Different letters indicate significant disparity from controls (*p* < 0.05). Data are mean ± SD (n = 3).

As shown in Figure 4, the phototactic behavior of *D. magna* exposed to 0.5 µg/L CIT was gradually inhibited during 6–24 h compared to a control group and was then followed by a marked reduction at 48 h. In contrast, under a CIT concentration of 10 µg/L, an important decrease in phototaxis was observed just after 6 h compared to the control and then a significant increase at 12 h with a continuously obvious decline. Comparatively, similar results were observed in former studies [26,51]. The study proved that the phototactic behavior of *D. magna* exhibited a gradual attenuation with increasing CIT concentration and exposure duration, which might subsequently impair the fundamental metabolic processes (including substrate energy metabolism) and disrupt neural signaling mechanisms [21,27]. At the molecular level, CIT as a selective serotonin reuptake inhibitor (SSRI) disrupts 5-HT neurotransmission critical for Daphnia’s phototaxis coordination. Chronic exposure (0.5 µg/L) may downregulate 5-HT1A receptors in the optic lobe; Qi et al. reported a responsive enhancement of negative phototactic behavior in *D. magna* under the 5-hydroxytryptamine 1A receptor antagonist exposure [52]. Acute high-dose exposure (10 µg/L) could induce oxidative stress, potentially damaging rhodopsin-containing photoreceptors. This is corroborated by Rivetti et al. [12], who documented a 40% reduction in Daphnia opsin gene expression under 50 µg/L fluoxetine [53]. The inhibition of phototaxis is mechanistically linked to the organism’s bioenergetic state. Photoreceptor cells, with their exceptionally high ATP demand, exhibit heightened sensitivity to metabolic perturbations. Initial phototaxis suppression may temporarily conserve ATP for critical phototransduction processes. However, chronic exposure ultimately depletes ATP reserves, thereby exacerbating photobehavioral deficits through energy-dependent pathway collapse. We hypothesize that the temporal dynamics of CIT-induced phototactic alterations in *D. magna* arise from a triphasic mechanism involving serotonergic disruption (0–6 h), metabolic compensation (6–12 h), and oxidative collapse (12–48 h). Therefore, the phototactic response intensity of *D. magna* can be utilized as a sensitive biomarker for assessing aquatic contamination levels or pollutant ecotoxicity. By employing phototactic behavior as a monitoring signal of the ecotoxicological effects of aquatic pollutants, potential hazards caused by pollution could be detected in a timely manner even when organisms begin to show minor initial effects.

#### 3.3.2. Feeding Behavior

**Figure 5:**
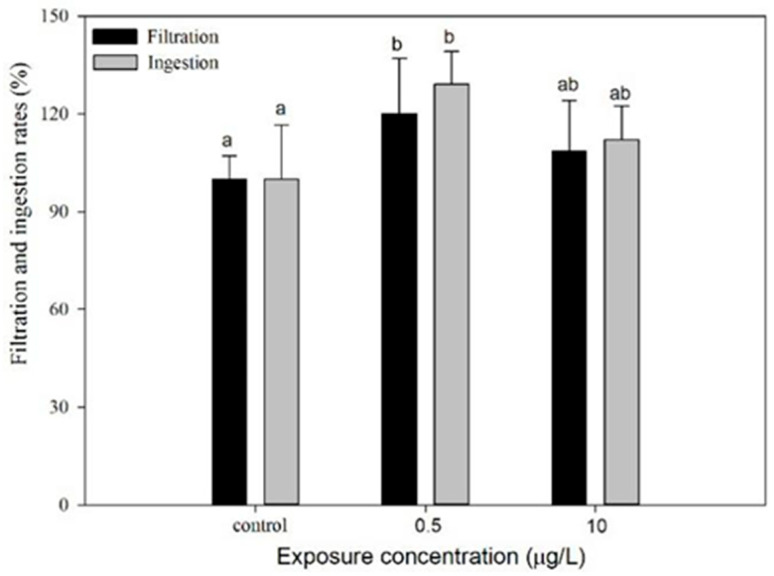
Dose-dependent effects of CIT on the filtration (F) and ingestion (I) rates of *D. magna* at 0.5 and 10 µg/L. Different letters indicate significant disparity from controls (*p* < 0.05). Data are mean ± SD (n = 3).

The outcomes indicated that the feeding behavior of *D. magna* exhibited induced responses to CIT exposure at the low concentration, while similar induction was observed at the high concentration, though this was not statistically significant (Figure 5). At 0.5 µg/L, the filtration rates increased by 32% compared to controls (*p* < 0.05). The result might be related to the acetylcholine (ACh) accumulation resulting from AChE inhibition. Mechanistically, CIT could indirectly influence ACh dynamics via serotonergic–cholinergic system cross-talk: serotonin accumulation enhances presynaptic ACh release, while competitive AChE inhibition prolongs synaptic ACh activity. The dual action—elevated ACh availability and delayed degradation—potentiates gut motility, thereby increasing both filtration and ingestion rates [54]. Comparatively, the stimulatory effects disappeared at 10 µg/L, likely because mitochondrial dysfunction counterbalanced the neurological stimulation. Thus, the ecological implications are two-fold. First, the *D. magna* may be temporarily enhanced at a CIT low exposure concentration of 0.5 μg/L, potentially reducing algal blooms [55]. However, population increases could destabilize food webs through excessive resource consumption. Second, chronic exposure at higher concentrations of 10 μg/L may impair energy metabolism, ultimately decreasing zooplankton populations and exacerbating eutrophication [36]. The 32% increase in filtration rate at 0.5 µg/L likely stems from acetylcholine (ACh) accumulation: 5-HT signaling enhances presynaptic ACh release while inhibiting acetylcholinesterase (AChE), prolonging ACh-mediated gut motility [26]. At 10 µg/L, however, the stimulatory effect vanished alongside a 40% decline in mitochondrial membrane potential [56], suggesting that metabolic collapse overrides neural excitation. This concentration-dependent pattern implies that low-dose CIT may transiently enhance algal grazing (benefiting water quality), but chronic high-dose exposure disrupts energy allocation, threatening zooplankton population sustainability [57].

### 3.4. Effect of CIT on the Antioxidant Enzyme Activities of D. magna

**Figure 6:**
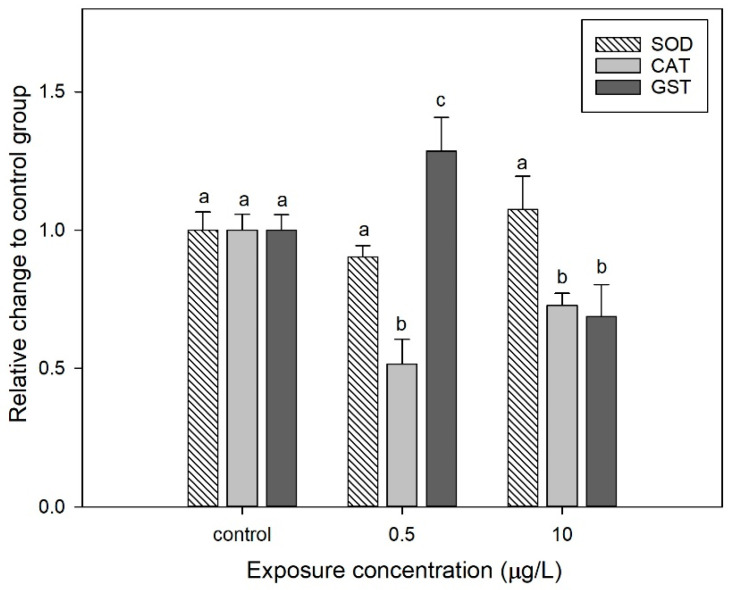
Changes in SOD, CAT, and GST enzyme activities at CIT exposure concentrations of 0.5 and 10 µg/L. Various letters indicate significant differences from controls (*p* < 0.05). Data are mean ± SD (n = 3).

At a low concentration (0.5 µg/L), CIT exposure significantly inhibited the CAT activity (49%) but induced the GST activity (58%) compare to control (Figure 6). The inhibition of CAT may stem from CIT directly interfering with enzyme active sites (e.g., binding to metal cofactors of Cu/Zn-SOD) or inducing excessive superoxide anion (O_2_^−^) production, leading to enzyme system overload [58]. The activation of GST is associated with the enhanced phase II detoxification pathways, reducing the toxicity of electrophilic pollutants through glutathione conjugation reactions [59]. Furthermore, the upregulation of GST may compensate for CAT functional defects, maintaining redox homeostasis. At a high concentration (10 µg/L), CIT exposure inhibited CAT (36%) and GST (24%), while SOD activity did not change significantly. Similarly, a previous study also found that different CIT concentrations exert multifaceted effects on *D. magna*. The drastic inhibition of CAT may reflect altered mitochondrial membrane permeability or oxidative damage triggered by H_2_O_2_ accumulation [60]. The decline in GST activity suggests the collapse of the detoxification system, leading to the accumulation of lipid peroxidation products such as MDA [61]. The stability of SOD may be related to the adaptive expression of enzyme subtypes (e.g., Mn-SOD) or the regulation of antioxidant genes through the Nrf2/Keap1 signaling pathway [62]. CIT initiates significant oxidative stress even at low concentrations, suggesting that its sublethal effects on aquatic invertebrates may have been underestimated. The results indicated that GST was a sensitive biomarker for monitoring CIT pollution levels in freshwater ecosystems.

### 3.5. Effect of CIT on the Gene Expression of D. magna

**Figure 7:**
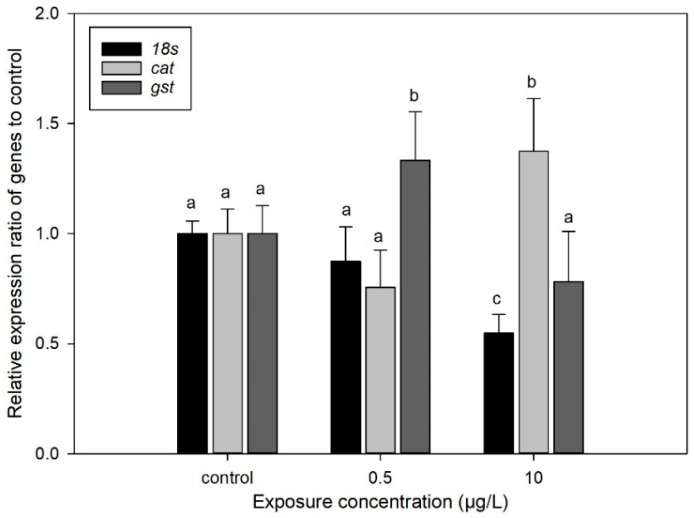
Changes in *18s*, *cat*, and *gst* gene expression at CIT exposure concentrations of 0.5 and 10 µg/L. Various letters indicate significant differences from controls (*p* < 0.05). Data are mean ± SD (n = 3).

As shown in Figure 7, the expression level of the *18s* gene was significantly reduced compared to the control at the CIT exposure concentration of 10 µg/L. Comparatively, the expression level of the *cat* gene was significantly increased compared to the control at a CIT exposure concentration of 10 µg/L, which likely represents an adaptive response to CIT-induced oxidative stress, such as hydrogen peroxide (H_2_O_2_) accumulation [62]. The *cat* gene expression was enhanced and CAT enzyme activity decreased by 36% at 10 µg/L, suggesting that post-transcriptional regulation may play a critical role in modulating enzyme functionality [60]. For the *gst* gene, the expression level was increased compared to the blank at 0.5 µg/L and slightly decreased at 10 µg/L. The *gst* gene induced its expression at 0.5 µg/L, consistent with a 58% increase in GST enzyme activity. This indicates that low CIT concentrations activate phase II detoxification pathways via the Nrf2/Keap1 signaling cascade, enhancing the glutathione-mediated conjugation and excretion of electrophilic metabolites. In contrast, at 10 µg/L CIT, *gst* expression was slightly suppressed, accompanied by a 24% reduction in GST activity. This collapse of detoxification capacity may result from oxidative damage to transcriptional machinery under high CIT stress [61]. The differential responses highlight concentration-dependent molecular adaptation mechanisms, providing critical molecular markers for assessing CIT-induced neurotoxicity.

### 3.6. Integrated Biomarker Response

**Figure 8:**
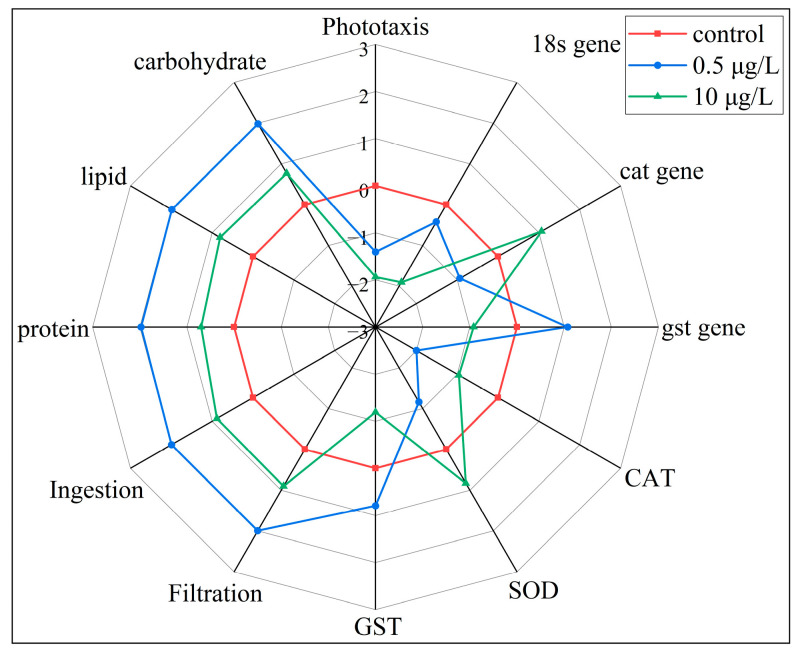
Star plot of IBR_v2_ index analyses in CIT-exposed *D. magna*. The area up to 0 reflects biomarker induction and the area down to 0 indicates biomarker inhibition.

The IBR_v2_ index was used to compare the comprehensive stress induced by various concentrations of CIT (Figure 8). IBR is a systematic and scientific method applied to several areas, as it allows for a visual comparison of toxic effects under different exposure conditions [63]. The numerical value of IBR_v2_ represents the total influence of a targeted compound on various indicators [64]. In our study, the IBR_v2_ values of *D. magna* exposed to CIT concentrations of 0.5 and 10 µg/L were 10.50 and 8.09. Similarly, the overall effect of carbamazepine on *D. magna* varies with the concentration of exposure in the previous studies, where low concentrations produced higher IBR_v2_ values. Compared with the 0.5 µg/L exposure group, the biomarkers *cat* gene, CAT, and SOD were induced in the 10 µg/L exposure group. Biomarkers such as phototaxis, *18s* gene, *gst* gene, GST, protein, lipid, carbohydrate, filtration, and ingestion were inhibited. The response of these biomarkers may imply their high sensitivity to CIT. This is consistent with the results of the analyses of individual indicators. The results of four indicators, *cat* gene, *gst* gene, SOD, and GST, were more representative, which helped us to better understand the toxicity of CIT to *D. magna*. Interestingly, the stress caused by CIT exposure is stronger at 0.5 μg/L. This may be due to the fact that CIT, at a concentration of 10 μg/L, triggers the defensive mechanism faster and reduces oxidative stress through the gene expression (*gst* gene, *cat* gene) of SOD, CAT, and GST enzymes. Subsequently, under this rapid response mechanism, the filtration and intake rates and available energy of *D. magna* exposed to 10 μg/L were less affected. Biomarkers may gradually adjust back to the baseline state after a short response time. Therefore, this made the value of IBR_v2_ in the 10 μg/L exposure group relatively low. Likewise, grass carp exposed to high concentrations of enrofloxacin activated defense mechanisms more rapidly and biomarkers gradually returned to baseline levels in the previous studies [65]. Future studies should explore more sensitive biomarkers to more accurately assess the impact of CIT on *D. magna*.

## 4. Conclusions

These findings suggest that antidepressants elicit ecotoxicity through oxidative stress pathways and complex mechanisms involving neurotransmitter-mediated interference (e.g., serotonin-regulated feeding behavior), cardiac function modulation, and nutrient metabolism disruption. This study demonstrated that CIT induces multifaceted toxic effects in *D. magna*, including bioaccumulation, behavioral alterations, and oxidative stress. CIT exhibited significant bioaccumulation potential in *D. magna*, with accumulated concentrations reaching 285.6 and 673.6 µg/kg at exposure levels of 0.5 and 10 µg/L. Concentration-dependent responses were observed where low-concentration CIT (0.5 µg/L) enhanced protein and lipid biosynthesis, and high-concentration exposure (10 µg/L) induced metabolic dysregulation and excessive lipid catabolism. Behavioral analyses revealed phototactic and feeding response modifications in *D. magna*. Phototactic behavior may serve as a sensitive biomarker for aquatic contamination. Short-term feeding enhancement might transiently inhibit algal blooms, but prolonged exposure risks disrupting population equilibrium. Oxidative stress responses further elucidated CIT’s dual effects in that low concentrations triggered a surge in GST activity, while the collapse of the antioxidant system at elevated concentrations potentially led to the accumulation of lipid peroxidation products. Future research should integrate transcriptomics (e.g., SOD/CAT gene expression) and metabolomics (e.g., the analysis of lipid peroxidation products) to reveal the multi-layered toxicity mechanisms of CIT, potentially employing non-target trend analysis for identifying the CIT parent and its transformation products. Future strategies should prioritize advanced treatment technologies (e.g., ozonation, membrane filtration) to mitigate CIT discharge and ensure the protection of aquatic ecosystems from emerging pharmaceutical contaminants. Additionally, the synergistic effects of CIT with co-occurring pollutants (e.g., microplastics, heavy metals) warrant investigation as combined exposures may exacerbate ecological risks.

## Figures and Tables

**Table 1:** The primer sequence of selected genes.

Genes	Forward Sequence	Reserve Sequence
β-actin	ACGATGATGTTGCGGCTTTG	CCGACAATGGAGGGGAAGAC
*18s*	CCTGAGAAACGGCTACCACATC	CTCGGAAGAGTCCCGTATCGT
*gst*	TCTATTATCCCATCATGTTCAGCG	CCAGCAGCATACTTGTTCTGACC
*cat*	ATCGCCTTGGAACAAACTACCT	AGCTGTTCGGGAAGTAATTTGG

## Data Availability

The original contributions presented in this study are included in the article. Further inquiries can be directed to the corresponding author.

## Supplementary Materials

The following supporting information can be downloaded at: https://www.mdpi.com/article/10.3390/toxics13070532/s1, Text S1. Mass spectrometry parameters for CIT determination. Text S2. The specific calculation method of integrated biomarker response version 2 (IBRv2). Table S1. Mobile phase compositions for the separation methods. Table S2. Optimized MS/MS parameters for the CIT.
