# Supplementary material for: The Effect of the Antidepressant Citalopram on the Bioconcentration and Biomarker Response of Daphnia magna at Environmentally Relevant Concentrations
Source: Toxics. 2025 Jun 25;13(7):532. doi: 10.3390/toxics13070532 (PMC12300989; doi:10.3390/toxics13070532)
Supplement: Supplementary file 1 [file toxics-13-00532-s001.zip › toxics-3667262-supplementary.pdf]

The Effect of the Antidepressant Citalopram on the Bioconcentration and Biomarker Response of *Daphnia magna* at Environmentally Relevant Concentrations

Haohan Yang *, Jiacheng Tan, Hanyu Jiang, Hao Xing, Jingnan Zhang, Dexin Kong, Zhuoyu Chen and Linghui Kong

College of Environmental Science and Engineering, Yangzhou University, Yangzhou 225127, China; 231601213@stu.yzu.edu.cn (J.T.); 231602108@stu.yzu.edu.cn (H.J.); mz120241359@stu.yzu.edu.cn (H.X.); 231601220@stu.yzu.edu.cn (J.Z.); 231601208@stu.yzu.edu.cn (D.K.); mz120231301@stu.yzu.edu.cn (Z.C.); mz120231312@stu.yzu.edu.cn (L.K.)

* Correspondence: hhy@yzu.edu.cn

Text lists

Text S1 Mass spectrometry parameters for CIT determination

Text S2 The specific calculation method of integrated biomarker response version 2 (IBR_{v2})

Table lists

Table S1. Mobile phase compositions for the separation methods

Table S2. Optimized MS/MS parameters for the CIT

Text S1 Mass spectrometry parameters for CIT determination

Ultrahigh-performance liquid chromatography was performed employing a Waters Acquity UPLC system (Milford, MA, USA) coupled to a Waters Xevo TQMS (Milford, MA, USA) working in ESI ionization mode. Separation of the CIT was achieved on a Waters Acquity UPLC BEH C18 column (1.7μm, 100 mm×1.7μm), kept at 40 °C, with two solvents: A (98:2, Water/Methanol+0.1% Formic acid) and B (Methanol). The flow was 0.4 mL/min, and the gradient profile was in Table S2. From both standard solutions and samples, kept at 10 °C, a volume of 5 μL was injected, after which the needle was rinsed with 750 μL of weak wash solution (water/methanol, 90:10) and 250 μL of strong wash solution (methanol/water, 90:10). Detection was performed by multiple reaction monitoring (MRM) with a Waters Xevo TQMS (Milford, MA, USA) triple quadrupole (QQQ) equipped with an electrospray (ESI) source. Capillary voltage was 3 kV; the source was kept at 150 °C; desolvation temperature was 500 °C; cone gas flow, 50 L/h; and desolvation gas flow, 900 L/h.

Text S2 The specific calculation method of integrated biomarker response version 2 (IBR_{v2})

Integrated biomarker response index (IBR_{v2} version) was analyzed for all measured biomarker data and calculated as follows:

Firstly, a log transformation was performed on all biomarker measurements:

$$Y_i = \log (X_i / X_0)$$

where X_i is the average value of each biomarker in an exposure and X_0 is the corresponding average value of each biomarker in control.

Secondly, the standardized biomarker value was calculated according to the following formula:

$$Z_i = (Y_i - \mu) / \sigma$$

where μ and σ represent the average and standard deviation of each biomarker across all exposure groups, encompassing the control group.

Thirdly, for every biomarker within each exposure group, the deviation (A_i) was calculated as:

$$A_i = Z_i - Z_0$$

where Z_0 signifies standardized valuation of the control group. The star plot of IBR_{v2} index analysis was drawn using the values of A_i.

Then, the deviation value was transformed as follows to ensure that the data ranged between -1 and 1:

$$S_i = A_i / \max (|A_i|)$$

where $\max (|A_i|)$ is the deviation index with the largest absolute value for each biomarker across all exposure groups.

Finally, the IBR_{v2} was obtained for each exposure group:

$$IBR_{v2} = \sum |S_i|$$

where $|S_i|$ represents the absolute value of the transformed deviation index.

Table S1. Mobile phase compositions for the separation methods

Time (min)	A% (98:2, Water/Methanol+0.1% Formic acid)	B% (Methanol)
0	95	5
0.2	95	5
3	2	98
4	2	98
4.01	95	5
5	95	5

Table S2. Optimized MS/MS parameters for the CIT

Chan Reaction	Dwell(secs)	Cone Volt.	Col.Energy	Delay (secs)	Compound	Formula|Mass Comments
325.10 > 108.93	0.025	38.0	26.0	Auto	Citalopram 324.2	IntelliStart Generated
